# A systematic review of the global prevalence and incidence of shoulder pain

**DOI:** 10.1186/s12891-022-05973-8

**Published:** 2022-12-08

**Authors:** J. Lucas, P. van Doorn, E. Hegedus, J. Lewis, D. van der Windt

**Affiliations:** 1 Primary Care Centre Versus Arthritis, School of Medicine, Keele University, Keele, Staffordshire UK; 2 Department of General Practice, Erasmus Medical Centre, Rotterdam, Netherlands; 3 Doctor of Physical Therapy Program, Tufts University School of Medicine, 101 E Washington Street, Suite 950, Phoenix, AZ 85004 USA; 4 Therapy Department, Central London Community Healthcare National Health Service Trust, London, UK; 5 Musculoskeletal Research, Clinical Therapies, University of Limerick, Limerick, Ireland

**Keywords:** Shoulder pain, Incidence, Prevalence, Epidemiology, Primary care, Global health

## Abstract

**Background and objectives:**

Studies reporting on the population burden of people living with shoulder pain show wide heterogeneity in terms of case definition, study samples, and occurrence. This systematic review aims to summarize evidence pertaining to the prevalence and incidence of shoulder pain, including variability based on sex and geography. We also explored the potential influence of methodological limitations and important sources of heterogeneity (case definition and reference period) on reported estimates of shoulder pain prevalence.

**Databases and data treatment:**

The study protocol was registered on Prospero under CRD42021243140. We searched EMBASE, CINAHL, Web of Science and Medline from inception to March 2021. Study selection, data extraction and risk of bias assessment was conducted by a team of three researchers. We performed a narrative synthesis of the data, using forest plots to summarize study findings, and stratified data presentation to explore the potential association of risk of bias, case definition, and reference period with estimates of prevalence and incidence of shoulder pain.

**Results:**

We obtained data from 61 studies reporting data from high-, middle- and low-income countries. The overall risk of bias was low, with most rated as “low-risk” and no studies rated as “high-risk”. The community prevalence of shoulder pain varied widely across the countries included in our review, with a median of 16% (range 0.67 to 55.2%). Longer reference periods were typically associated with higher prevalence estimates. Primary care prevalence ranged from 1.01 to 4.84% (median 2.36%). Estimates were generally higher for women than men and were higher in high-income nations. The incidence of shoulder pain ranged from 7.7 to 62 per 1000 persons per year (median 37.8 per 1000 persons per year). Risk of bias did not clearly explain variability in study findings, but there was considerable variation in study samples, methods used, and a relative absence of data from low-income countries.

**Conclusions:**

Our review demonstrates that a significant proportion of the population across the world will experience shoulder pain daily, yearly, and throughout a lifetime. Regional gaps in evidence and methodological inconsistencies must be addressed in order to establish a more definitive global burden.

**Supplementary Information:**

The online version contains supplementary material available at 10.1186/s12891-022-05973-8.

## Introduction

After low back pain and knee pain, shoulder pain has been estimated to be the third most common musculoskeletal presentation in primary care [1]. The prognosis for people presenting with musculoskeletal shoulder pain varies widely between individuals with, on average, 50% of people with shoulder pain still reporting symptoms 6 months after presenting in primary care [2]. In addition to pain, functional disabilities are common and can interfere with work, hobbies, social, and sporting activities and may also be associated with psychological distress and reduced quality of life [3]. Shoulder pain consequences generate high costs to society. A cost-estimation study performed in Sweden reported the financial burden associated with shoulder pain as €4139 per patient, with sick leave accounting for more than 80% of the total costs [4].

The global burden of shoulder pain, in terms of incidence and prevalence, has been assessed previously by Luime et al. [5]. Their review incorporated 18 studies reporting on the prevalence and one study reporting incidence of shoulder pain in the population, based on data collected until 2001 in 9 different countries. Results, however, vary greatly, possibly related to differences in source populations (geographic location, economic differences, variability in healthcare setting), study samples (e.g., sex distribution), case definition (e.g. shoulder pain exclusively), or methodological limitations (risk of bias). These inconsistencies challenge our understanding of the burden of shoulder pain across countries and populations. Furthermore, there has been an increase in shoulder pain conditions reported [6] and the body of evidence regarding the burden of shoulder pain has substantially increased since Luime et al’s [5] review. New data are now available from additional countries, and from community as well as primary care settings, where most people with shoulder pain are being managed. Therefore, an update on the global burden of shoulder pain is warranted.

Our review aims to summarize existing evidence regarding the population and primary care burden of shoulder pain, with the following objectives: (1) to summarize estimates of the prevalence and incidence of shoulder pain, including variability related to source population (community or primary care), sex and economic status; (2) to provide insight into the methodological strengths and limitations of available research; and (3) to explore the potential impact of two main sources of heterogeneity (the use of different case definitions and different reference periods) on estimates of shoulder pain prevalence.

## Methods

### Prospero protocol

The title and protocol for this systematic review was registered on PROSPERO, ID: CRD42021243140. The review has been reported according to the Preferred Reporting Items for Systematic Reviews and Meta-Analyses (PRISMA).

### Eligibility criteria

We included observational studies that reported incidence and/or prevalence of shoulder pain in adults in the community or in primary care settings. Cohort designs were required to estimate incidence. To be included, studies were required to use a case definition that was restricted to the shoulder (i.e., not also incorporating neck or upper limb pain). We excluded studies reporting on- shoulder pain related to acute trauma, inflammatory or other specific musculoskeletal conditions involving the shoulder, and studies only reporting on persistent shoulder pain (with a minimum duration of 3 months). We included studies reporting on sample populations of a minimum of 100 people to reduce the risk of extracting estimates with low precision. We restricted our search to articles published in the English language. When multiple publications were identified reporting on the same sample, we avoided using data from the same population sample multiple times in the analysis: if estimates for multiple time-points were given, we extracted the most recent estimate; and if estimates for a range of case definitions or reference periods were given, we extracted estimates that were most similar to those used by the majority of studies, aiming to reduce heterogeneity.

### Searching and selection

Searches were conducted using the Medline, EMBASE, Web of Science and CINAHL databases, from inception until 22/3/21. Searches were designed with advice from an information specialist, using search terms (Medical Subject Headings and text words) related to shoulder pain and specific shoulder conditions, terms to identify epidemiological studies, and terms to identify primary care and community settings. The full search strategy is included in the supplementary files (Supplementary Fig. 1). A substantial number of studies were conducted as part of the World Health Organisation COPCORD (Community Oriented Program for Control of Rheumatic Diseases) initiatives. Therefore, the COPCORD website (www.copcord.org) was checked to ensure all relevant studies were identified.

An initial screening based on study title was conducted by JLu. This was followed by two-person screening based on abstract and full text, in which each study was assessed by two screeners independently (two of JLu, PvD, DvdW). Any conflicts were reviewed by the 3rd reviewer and discussed where needed to achieve consensus.

### Data extraction and methodological appraisal

A customised data extraction instrument was developed for the review, and pilot-tested by the team. Data extraction was conducted by one reviewer, and checked by a second reviewer, with inconsistencies identified and resolved by discussion. Data extracted included: study setting and design characteristics; sample demographics; case definition; number of subjects at risk; number of cases with shoulder pain; prevalence or incidence reference period; and estimates of prevalence or incidence with 95% confidence interval. Income group for each country was obtained from the World Bank 2021–2022 country classification by income (https://datahelpdesk.worldbank.org/knowledgebase/articles/906519-world-bank-country-and-lending-groups). If standard error (SE) or 95% confidence intervals were not provided with the prevalence estimate (p), the SE was calculated using the formula SE = [(p x (1-p))/n] where n is the sample size. For incidence estimates reported without 95% confidence intervals, these were derived using the Mid-P exact test accessed through www.openepi.com/PersonTime1/PersonTime1.htm.

The methodological quality of each of the studies was assessed using the checklist for prevalence studies developed by Hoy et al. [7], which includes 10 items to assess risk of bias related to population sampling and response; case definition and methods used to assess shoulder pain; and data analysis and reporting. Each item was rated as indicating low, high or unclear risk of bias, if the article included insufficient information to enable an assessment of risk of bias. Results are presented graphically for each individual study, and cumulatively across bias domains to summarize strengths and limitations of previous research. Risk-of-bias figures were created using the “robvis” R package [8]. In addition, we used guidance by Hoy et al. [7] to calculate a summary score for each study reflecting overall risk of bias, where 7–10 items scored as “high risk” indicated an overall high risk of bias,, 4–6 items an overall moderate risk, and 0–3 items scored an overall low risk of bias. These summary scores were used to describe overall risk of bias for each individual study and generate awareness of risk of bias when interpreting their results.

### Evidence synthesis

#### Objective 1

Data on prevalence and incidence were separately tabulated for studies conducted in community/general population settings and for primary care-based studies. When studies presented prevalence estimates stratified for sex, these were extracted and presented in a linked forest plot. Furthermore, the countries where the study was performed were stratified based on income group. Meta-analysis was not appropriate, given the wide heterogeneity in source populations, study samples, case definitions, and reference periods. Therefore, estimates were presented graphically and summarized narratively. Forest plots were generated using Microsoft Excel™ [9].

#### Objective 2

To provide insight into the risk of bias and to determine if our study findings differ from others (associated with higher risks and limitations), we present a summary of findings table. This table summarizes the median and range of prevalence and incidence estimates for subgroups of studies classified as having low, medium, or high overall risk of bias.

#### Objective 3

We explored the influence of studies using different case definitions or different reference periods across studies, by presenting median and range of prevalence estimates in the summary of findings table, stratified by reference period (1 week or less; 2 to 6 weeks; 12 months or longer), and stratified by different types of case definition (studies using the COPCORD definition; studies using a more specific case definition, mostly based on frequency or duration of shoulder symptoms; and studies identifying cases of shoulder pain using health records). We graphically illustrated the potential impact of variability in reference period in a forest plot, with prevalence estimates ordered based on the reference period used.

## Results

### Search results

**Fig. 1:**
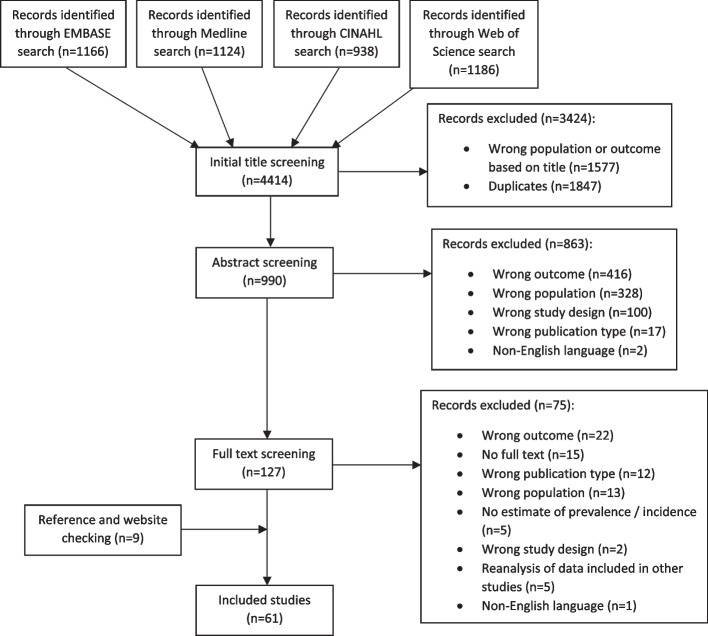
Summary of searches and results of screening

Our search identified 4414 publications, and after the screening process 61 studies were included in the review, providing 61 prevalence estimates and 8 incidence estimates (See Fig. 1 for a summary of study selection process).

### General study characteristics

Of the 61 included studies, 27 were from Europe, 15 from Asia, 7 from South America, 7 from North and Central America, 3 from Australasia, and 1 from Africa. Studies from the United Kingdom (UK) were the most common. Twenty-five studies reported separately for biological sex. The majority (84%) of studies used a survey/questionnaire or interview method to collect data on shoulder pain, with the remainder analysing primary care health records. Studies were published between 1991 and 2020 with 93% published after 2000. No studies were excluded based on methodological quality. Supplementary Tables 1 and 2 summarise the characteristics of each study, with studies grouped according to three broad categories of case definition. The main findings for each of the review objectives are summarized in Table 1.

**Table 1:** Summary of findings of estimates of the prevalence and incidence of shoulder pain

	Prevalence (%)		Incidence (per 1000 person-years)^a^	
	Number of estimates	Median (range)	Number of estimates	Median (range)
**Overall**	61	14.5 (0.67 to 55.2)	8	37.8 (7.7 to 62)
*Setting*				
Primary care	7	2.36 (1.01 to 4.84)	6	37.8 (7.7 to 29.5)
Community/general population	54	16.0 (0.67 to 55.2)	2	11.4; 62
*Sex*				
Women	25	20.2 (2.0 to 62.3)		
Men	25	12.5 (2.1 to 46.6)	–	–
*Economic status*				
High income nations	35	16.9 (1.01 to 55.2)		
Upper-middle income nations	12	8.0 (3.56 to 24.0)		
Lower-middle income nations	10	9.5 (2.0 to 22.7)		
Low income nations	1	0.67	–	–
*Risk of bias (overall score)*				
Low	44	21 (1.01 to 42.4)		
Moderate	17	10.34 (0.67 to 55.2)	–	–
*Case definition*				
COPCORD definition^b^	23	7.4 (0.67 to 22.7)		
Definition similar to COPCORD	19	21.0 (9.0 to 55.2)		
Definition requiring minimum symptom frequency/duration	12	20.49 (3.06 to 30.7)		
Primary health care records	7	2.36 (1.01 to 4.84)	–	–
*Reference period*				
Point prevalence	3	21 (20.9 to 26)		
7-day prevalence	24	8.6 (2.0 to 34.2)		
2 to 6-week prevalence	9	21 (11.7 to 42.4)		
12-month prevalence	17	16.0 (1.01 to 55.2)		
Lifetime	1	22.3		

^a^No further subgroup analyses are presented for incidence estimates, given the small number of studies providing data on incidence

^b^COPCORD definition: Pain, stiffness or swelling in the shoulder region. Marked on body map

### Objective 1: prevalence and incidence of Shoulder pain

**Fig. 2:**
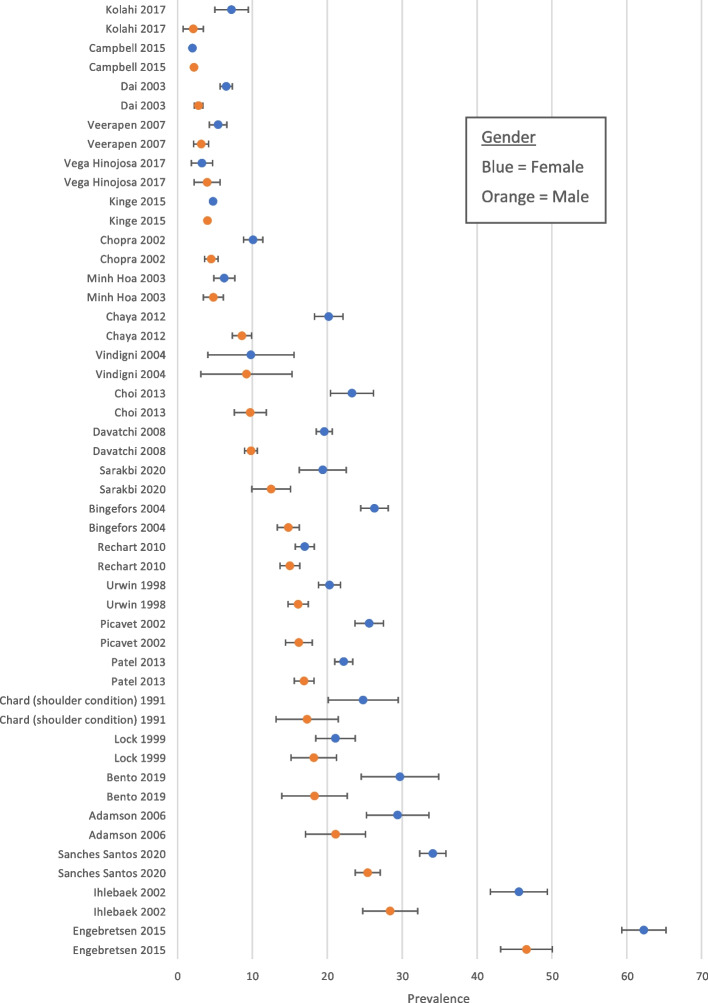
Forest plot showing gender-specific prevalence estimates

A total of 7 studies reported the overall prevalence of shoulder pain in the primary care setting, with estimates ranging from 1.0 to 4.8% (median 2.4%). In general population (community-based) samples estimates varied widely, from 0.7 to 55.2% (median 16.0%). Prevalence was generally higher in women than in men (data from 25 studies, see Fig. 2), and high-income nations had higher median prevalence (16.9%, range 1.0 to 55.2) compared to upper-middle income (8.0%, range 3.6 to 24.0%), lower-middle income (9.5%, range 2.0 to 22.7%) and low-income (0.7%) countries.

Estimates of the incidence of shoulder pain (8 studies) ranged from 7.7 to 62.0 per 1000 persons per year, with 7 of the 8 estimates derived from studies conducted in high income countries. Only two of the studies were conducted in general population samples, reporting an incidence of shoulder pain of 11.4 per 1000 person-years (Kuwaiti nationals [10]) and 62 per 1000 person-years (residents of villages near Dhaka, Bangladesh [11]).

### Objective 2: methodological quality

Supplementary Figs. 2 and 3 summarise the results of risk of bias assessment of all studies included in the review, separately for studies reporting on prevalence (Sup fig. 2) and incidence (Sup fig. 3). Based on the summary score, 40 out of the 56 studies reporting on prevalence were rated as “low risk” of bias, with the remainder rated as “moderate risk”, and 7 out of the 8 of studies reporting on incidence were rated as “low risk” with the remainder rated as “moderate risk”. Summary of findings Table 1 shows that there still is wide remaining variability in prevalence estimates between these two subgroups of studies, indicating overall study quality does not explain variability in prevalence. The most frequently occurring risk of bias amongst prevalence studies was that the target population was not considered representative of the national population (item 1, 35 out of 56 (63%) studies). In 23 (41%) of the prevalence studies the risk of response bias (item 4) was considered high or unclear. For incidence studies the most common risk of bias was that data were not collected directly from participants (5 out of 8 (63%) studies). Across studies, there were frequently issues with data presentation; numerators or denominators (item 10) were unclear or considered inappropriate in 32 out of the 61 studies (52%).

### Objective 3: potential influence of main sources of heterogeneity on prevalence estimates

#### Case definition

**Fig. 3:**
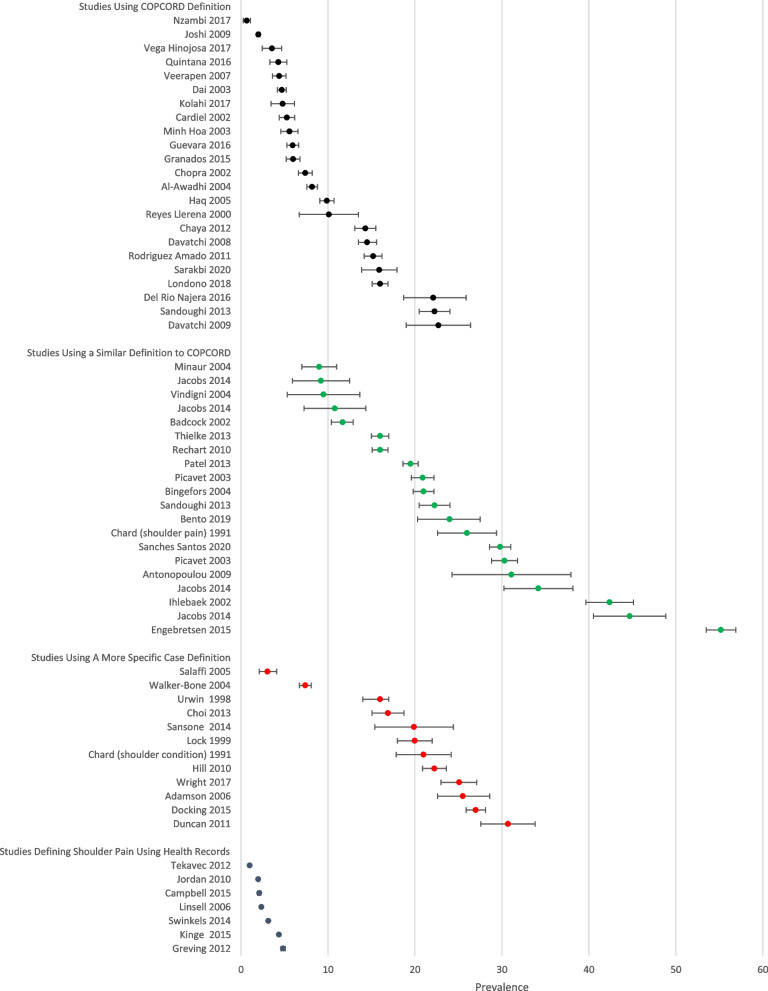
Forest plot showing prevalence estimates, arranged by case definition

Data extraction identified variability in the definition used to identify people with shoulder pain: Twenty-three of the included studies were conducted as part of the COPCORD programme, all using the same methods for case definition and data ascertainment. Twelve studies used a more restrictive case definition often including a minimum duration or frequency of shoulder pain (see Table 1). Figure 3, which presents prevalence estimates grouped according to case definition shows that studies using a more restrictive or specific case definition generally produced similar prevalence estimates. The 7 studies using primary healthcare records to identify cases with shoulder pain produce consistently lower estimates of shoulder pain prevalence, as expected.

#### Reference period

**Fig. 4:**
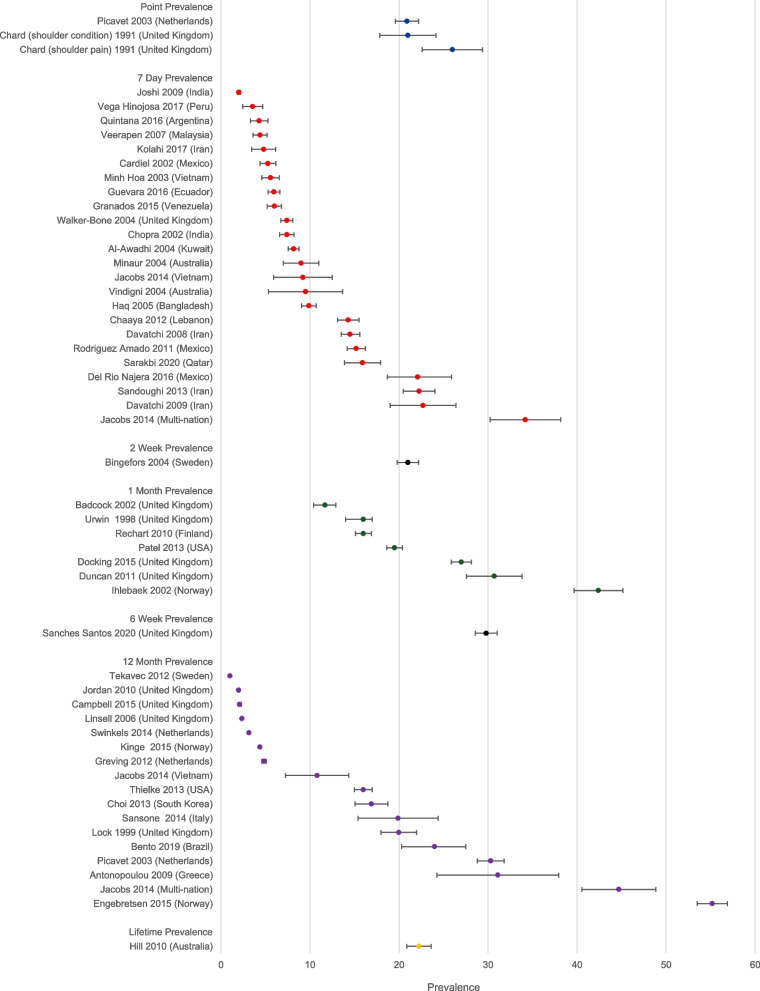
Forest plot showing prevalence estimates, arranged by reference period

The majority of studies focused on participants experience of shoulder pain in the past week. Others used reference periods of up to 12 months or even lifetime. Figure 4 indicates that, where the reference period was clearly described, a longer duration of the reference period was generally associated with increasing estimates of prevalence. There was however substantial remaining variability even amongst studies using the same reference period.

## Discussion

### Summary of findings

Our review included 61 studies published between 1991 and 2020 that reported prevalence and/or incidence rates. In survey-based studies, we found prevalence rates ranging from 10.8–55.2% for a reference period of 12-months or more, 11.7–42.4% for a reference period of 2–6 weeks, 2.0–34.2% for a reference period of 7-days, and 20.9–26% for point-prevalence. The prevalence estimates when primary care health records were used for data ascertainment ranged from 1.0 to 4.8%. The annual incidence rates varied from 7.7–62 per 1000 person years. Women were more likely to report shoulder pain than men, and studies conducted in higher income countries generally produced higher prevalence estimates than those from lower income countries.

It can be expected that the reference period has a considerable effect on the reported prevalence rates. A longer reference period could lead to higher prevalence rates. However, because the prevalence ranges are so large even within a certain reference period there was a considerable overlap in prevalence results between reference periods. Therefore, based on the results of this review it cannot be concluded that the reference period has an effect on the prevalence rate. A possible explanation of the large ranges found between studies with the same reference period could be the differences in used case definition. However, our findings suggest that even between studies using the same case definition and the same reference period (studies performed in the COPCORD program) the differences in prevalence rates are still substantial. For these studies the main reason for differences in reported prevalence rates appear to be due to one or more differences in study population.

Using Mexico as an example, two studies [12, 13] were conducted as part of the COPCORD project, using the same sampling methods, data ascertainment method, and reference period, yet produced very different prevalence estimates (Cardiel et al.: 5.28%; Rodriguez Amado et al.: 15.2%). The significant difference between these studies is the study population, with Cardiel et al. recruiting subjects from an urban community in Mexico City, and Rodriguez Amado et al. recruiting from a mixed urban and rural community in Nuevo Leon in North-eastern Mexico. Similar differences in prevalence were observed in studies conducted in Iran [14–16].

Several factors which were outside the scope of this review, such as age, occupation, presence of co-morbid health conditions, and even health literacy levels and access to healthcare may influence the likelihood of people reporting musculoskeletal pain. In particular, the relationship between age, occupation and musculoskeletal pain is complex, and we were not able to explore this in our review due to limitations in the available data. The prevalence of shoulder pathology increases linearly with increasing age, however the prevalence of pain appears to decrease after the age of 65 [17]. This could be explained by the impact of physical activity and occupation on shoulder pain, a relationship which has been demonstrated elsewhere [18]. The global population is getting older and many countries are increasing their retirement age despite evidence that healthy working life expectancy is not increasing at the same rate [19]. It is likely therefore that the burden of shoulder pain problems in working adults will increase over time. Future epidemiological studies will need to consider these factors.

### Strengths of our review

The main strength of our review is the expansion of included studies over the previous review. Eighty per cent of the included studies were conducted after the most recent review of shoulder pain prevalence in 2004, and we obtained significantly more estimates from South America, North America, Asia, and Australasia. This represents a significant expansion of available data compared to the most recent review [5].

We also conducted an extensive search of several databases and used a team of three reviewers to verify study selection and data extraction.

### Limitations of our review

The first potential limitation of our review is that, as with all systematic reviews, our search protocol may not have captured all available studies. We created a thorough search protocol and applied it in four major publication databases, however we did have to keep the terms of the search sufficiently narrow to prevent an overwhelming number of results that would have been impractical to work with.

We also decided to exclude non-English language studies. This is often reported as a weakness of studies as it introduces a potential source of bias. However, in our case only 3 studies were excluded based on publication language alone, and our search was sufficiently broad to capture data from many non-English speaking countries. Furthermore, there is evidence that exclusion of non-English language papers from systematic reviews may not have a significant impact on the overall conclusions [20].

The final limitation of our review was that we were not able to conduct a meta-analysis. This was due to the heterogeneity among the included studies which we have discussed above. Instead, we opted for a narrative synthesis of the results.

### Limitations of the included studies

There were 45 studies with an overall low risk of bias and 16 studies with an overall moderate risk of bias. None of the included studies had a high overall risk of bias. The most frequent limitation across the included studies was the use of a target population that was not representative of the national population. This was particularly problematic in countries where the socioeconomic conditions varied considerably across the country, such as between urban centres and rural areas.

There was also a lack of standardised case definitions and data ascertainment methods. Many studies utilised the COPCORD questionnaire or the Nordic musculoskeletal questionnaire but the application of these was not always consistent across studies.

Several studies did not obtain an adequate response rate leaving them at more risk of selection bias, and in over half of studies there were issues with data presentation, mainly not reporting numerators and denominators with the prevalence or incidence estimate.

### Implications for practice and research

Overall, this review shows that there are substantial differences in reported prevalence and incidence rates of shoulder pain. These differences could be large and explained by variations in research methods used in these studies. We found differences in definitions of shoulder pain, data ascertainment, and use of prevalence and/or incidence period which undeniably led to divergence in results. These differences underline the importance of a standardized research methodology to compare epidemiologic study results and allow for clear interpretation.

Interestingly, studies performed under the COPCORD program with a standardized research methodology showed considerable differences in reported prevalence even within a country. This variance even with standardized methodology supports the evidence that the study population has a considerable influence on the estimated prevalence of shoulder pain.

Healthcare workers and policy makers should be aware of the variations in research methods used and the population examined when interpreting prevalence or incidence rates reported by a single study and take caution when generalizing these results to other populations.

## Supplementary Information

**Additional file 1: Supplementary Table 1.** Study characteristics and results of prevalence studies, grouped by case definition

**Additional file 2: Supplementary Table 2.** Study characteristics and results of incidence studies

**Additional file 3: Supplementary Fig. 1.** Database search strategy

**Additional file 4: Supplementary Fig. 2.** Risk of bias scores prevalence studies

**Additional file 5: Supplementary Fig. 3.** Risk of bias scores incidence studies

## Data Availability

The datasets used and/or analysed during the current study available from the corresponding author on reasonable request.
