# Supplementary material for: A systematic review of the global prevalence and incidence of shoulder pain
Source: BMC Musculoskelet Disord. 2022 Dec 8;23:1073. doi: 10.1186/s12891-022-05973-8 (PMC9730650; doi:10.1186/s12891-022-05973-8)
Supplement: Supplementary file 1 — Additional file 1: Supplementary Table 1. Study characteristics and results of prevalence studies, grouped by case definition [file 12891_2022_5973_MOESM1_ESM.docx]

**Studies using COPCORD definition (or very similar)**

| **Study** | **Year** | **Country** | **Data ascertainment** | **Study setting** | **Sample Size** | **Study population** | **Case definition** | **Prevalence period** | **Prevalence** | **Risk of Bias Summary Score** |
| --- | --- | --- | --- | --- | --- | --- | --- | --- | --- | --- |
| Al-Awadhi (1) | 2004 | Kuwait | Face-to-face questionnaire | Community | 7670 | Age 15+, Kuwaiti nationals | Pain, stiffness or swelling in the shoulder region. Marked on body map | 7 days | 8.17% | Low |
| Cardiel (2) | 2002 | Mexico | Face-to-face questionnaire | Community | 2500 | Age 18+. Urban. Residents of Mexico city | Pain, stiffness or swelling in the shoulder region. Marked on body map | 7 days | 5.28% | Low |
| Chaaya (3) | 2012 | Lebanon | Face-to-face questionnaire | Community | 3530 | Age 15+ | Pain, stiffness or swelling in the shoulder region. Marked on body map | 7 days | 21% | Low |
| Chopra (4) | 2002 | India | Face-to-face questionnaire | Community | 4092 | Age 15+. Rural. Residents of Bhigwan region | Pain, stiffness or swelling in the shoulder region. Marked on body map | 7 days | 7.40% | Low |
| Dai (5) | 2003 | China | Face-to-face questionnaire | Community | 6584 | Age 15+. Urban. Residents of Shanghai | Pain, stiffness or swelling in the shoulder region. Marked on body map | Unclear | 4.7% | Low |
| Davatchi (6) | 2008 | Iran | Face-to-face questionnaire | Community | 10291 | Age 15+. Urban. Residents of Tehran | Pain, stiffness or swelling in the shoulder region. Marked on body map | 7 days | 14.50% | Low |
| Davatchi (7) | 2009 | Iran | Face-to-face questionnaire | Community | 1565 | Age 15+. Rural. Residents of Tuyserkan county | Pain, stiffness or swelling in the shoulder region. Marked on body map | 7 days | 22.70% | Low |
| Del Rio Najera (8) | 2016 | Mexico | Face-to-face questionnaire | Community | 1006 | Age 18+. Urban. Residents of Chihuahua | Pain, stiffness or swelling in the shoulder region. Marked on body map | 7 days | 22.1% | Moderate |
| Guevara (9) | 2016 | Ecuador | Face-to-face questionnaire | Community | 4877 | Age 18+. Urban. Residents of Cuenca living at their current address for at least 6 months | Pain, stiffness or swelling in the shoulder region. Marked on body map | 7 days | 5.95% | Low |
| Granados (10) | 2015 | Venezuela | Face-to-face questionnaire | Community | 3973 | Age 18+. Urban. Residents of Monogas State | Pain, stiffness or swelling in the shoulder region. Marked on body map | 7 days | 6.0% | Low |
| Haq (11) | 2005 | Bangladesh | Face-to-face questionnaire | Community | 5160 | Age 15+ | Pain, stiffness or swelling in the shoulder region. Marked on body map | 7 days | 9.88% | Low |
| Joshi (12) | 2009 | India | Face-to-face questionnaire | Community | 8145 | Age 15+. Rural. Residents of Pune. | Pain, stiffness or swelling in the shoulder region. Marked on body map | 7 days | 2.0% | Low |
| Kolahi (13) | 2017 | Iran | Face-to-face questionnaire | Community | 952 | Age 35-70. Urban. Permanent residents of Khamene city. | Pain, stiffness or swelling in the shoulder region. Marked on body map | 7 days | 4.80% | Low |
| Londono (14) | 2018 | Colombia | Face-to-face questionnaire | Community | 6693 | Age 18+. Living in the household for more than 2 years. | Pain, stiffness or swelling in the shoulder region. Marked on body map | Unclear | 16% | Low |
| Minh Hoa (15) | 2003 | Vietnam | Face-to-face questionnaire | Community | 2119 | Age 16+. Urban. Residents of Hanoi | Pain, stiffness or swelling in the shoulder region. Marked on body map | 7 days | 5.57% | Low |
| Nzambi (16) | 2017 | Congo | Face-to-face questionnaire | Community | 1500 | Rural. Residents of Gombe-Matadi area. | Pain, stiffness or swelling in the shoulder region. Marked on body map | Unclear | 0.67% | Low |
| Quintana (17) | 2016 | Argentina | Face-to-face questionnaire | Community | 1656 | Age 18+. Urban. Qom and resident for at least 6 months in Rosario | Pain, stiffness or swelling in the shoulder region. Marked on body map | 7 days | 4.29% | Low |
| Reyes Llerena (18) | 2000 | Cuba | Face-to-face questionnaire | Community | 300 | Adults. Urban. Residents of Santa Catalina | Pain, stiffness or swelling in the shoulder region. Marked on body map | Unclear | 10.10% | Moderate |
| Rodriguez Amado (19) | 2011 | Mexico | Face-to-face questionnaire | Community | 4713 | Age 18+. Residents of Neuvo Leon | Pain, stiffness or swelling in the shoulder region. Marked on body map | 7 days | 15.2% | Low |
| Sandoughi (20) | 2013 | Iran | Face-to-face questionnaire | Community | 2100 | Age 15+. Residents of Zahedan for more than 6 months | Pain, stiffness or swelling in the shoulder region. Marked on body map | 7 days | 22.26% | Low |
| Sarakbi (21) | 2020 | Qatar | Face-to-face questionnaire | Community | 1239 | Age 15+. Residents of Qatar | Pain, stiffness or swelling in the shoulder region. Marked on body map | 7 days | 15.9% | Low |
| Veerapen (22) | 2007 | Malaysia | Face-to-face questionnaire | Community | 2594 | Age 15+. Semirural. Residents of Banting | Pain, stiffness or swelling in the shoulder region. Marked on body map | 7 days | 4.39% | Low |
| Vega Hinojosa (23) | 2018 | Peru | Face-to-face questionnaire | Community | 1095 | Age 18+. Residents of Juliaca and lake Titicaca who were born in region and lived there for >1 year | Pain, stiffness or swelling in the shoulder region. Marked on body map | 7 days | 3.56% | Low |

**Studies using a similar definition to COPCORD**

| **Study** | **Year** | **Country** | **Data ascertainment** | **Study setting** | **Sample Size** | **Study population** | **Case definition** | **Prevalence period** | **Prevalence** | **Risk of Bias Summary Score** |
| --- | --- | --- | --- | --- | --- | --- | --- | --- | --- | --- |
| Antonopoulou (24) | 2009 | Greece | Face-to-face questionnaire | Community | 176 | Age 20-75. Rural. Residents of Crete. | Ache, pain , discomfort, numbness in shoulder region. Marked on body map | 12 months | 31.10% | Low |
| Badcock (25) | 2002 | England | Face-to-face questionnaire | Community | 2606 | Age 18-75. | Ache or pain in the shoulder area. Marked on body map | 1 month | 11.7% | Low |
| Bento (26) | 2019 | Brazil | Face-to-face questionnaire | Community | 600 | Age 20+. Urban. Residents of Bauru | Pain located at a restricted area in or around the shoulder complex | 12 months | 24% | Low |
| Bingefors (27) | 2004 | Sweden | Face-to-face questionnaire | Community | 4506 | Age 20-64. Residents of Uppland County | Self-reported shoulder ache | 2 weeks | 21% | Moderate |
| Chard (28) | 1991 | England | Face-to-face questionnaire and physical examination | Community | 644 | Age 70+. Residents of Cambridge | Shoulder pain or disability | Point | 26% | Moderate |
| Engebretsen (29) | 2015 | Norway | Face-to-face questionnaire | Community | 1443 | Age 20-73. Urban. Residents of Ullensaker | Shoulder pain. Marked on body map | 12 months | 55.20% | Low |
| Ihlebaek (30) | 2002 | Norway | Face-to-face questionnaire | Community | 1240 | Age 15+ | Shoulder pain as assessed by the subjective health complaints inventory | 1 month | 42.40% | Moderate |
| Jacobs (31) | 2014 | Vietnam | Face-to-face questionnaire | Community | 295 | Age 20-88. Rural. Residents of Ben Tre Province | Pain or discomfort in the shoulder | 12 months | 10.8% | Low |
| Jacobs (31) | 2014 | Vietnam | Face-to-face questionnaire | Community | 295 | Age 20-88. Rural. Residents of Ben Tre Province | Pain or discomfort in the shoulder | 7 days | 44.7% | Low |
| Jacobs (31) | 2014 | Peru, Ecuador, Argentina | Face-to-face questionnaire | Community | 522 | Age 18+. Rural. | Pain or discomfort in the shoulder | 12 months | 9.2% | Low |
| Jacobs (31) | 2014 | Peru, Ecuador, Argentina | Face-to-face questionnaire | Community | 522 | Age 18+. Rural. | Pain or discomfort in the shoulder | 7 days | 34.2% | Low |
| Minaur (32) | 2004 | Australia | Face-to-face questionnaire | Community | 847 | Age 15+. Aboriginals resident in North Queensland. | Pain in the shoulder joint | 7 days | 9% | Low |
| Patel (33) | 2013 | USA | Face-to-face questionnaire | Community | 7601 | Age 65+. Not living in nursing homes | Pain in the shoulder | 1 month | 19.50% | Moderate |
| Picavet (34) | 2003 | the Netherlands | Postal questionnaire | Community | 3664 | Age 25+. | Pain in the shoulder | Point | 30.3% | Moderate |
| Picavet (34) | 2003 | the Netherlands | Postal questionnaire | Community | 3664 | Age 25+. | Pain in the shoulder | 12 months | 20.9% | Moderate |
| Rechardt (35) | 2010 | Finland | Face-to-face questionnaire | Community | 6237 | Age 30+. Excluding clinically diagnosed rheumatic arthritis and a positive rheumatoid factor. | Shoulder pain, ache or motion tenderness. Marked on body map | 1 month | 16% | Low |
| Sanchez Santos (36) | 2020 | England | Face-to-face questionnaire | Community | 5409 | Age 65+. Non-institutionalized, no terminal illness of less than 6 months or severe health or social concerns elderly resident in Merseyside, West-Yorkshire, West Midlands, Cambridgeshire, Gloucestershire, Dorset, Wiltshire, London or Oxfordshire. | Any trouble (ache, pain, discomfort) in the shoulder | 6 weeks | 29.80% | Low |
| Sandoughi (20) | 2013 | Iran | Face-to-face questionnaire | Community | 2100 | Age 15+. Residents of Zahedan for more than 6 months | Pain, stiffness or swelling in the shoulder region. Marked on body map | 7 days | 22.26% | Low |
| Thielke (37) | 2012 | USA | Face-to-face questionnaire | Community | 5093 | Age 65+. Non-institutionalized, ambulatory, not receiving radiation or chemotherapy for cancer, and expected to live in the area for 3 years | Pain in the shoulders | 12 months | 16% | Moderate |
| Vindigni (38) | 2004 | Australia | Face-to-face questionnaire | Community | 189 | Age 15+. Rural. Aboriginals resident in Kempsey district. | Ache, pain or discomfort in one or both shoulders | 7 days | 9.50% | Low |

**Studies using a more specific case definition for shoulder pain, and/or including a minimum symptom duration in their definition**

| **Study** | **Year** | **Country** | **Data ascertainment** | **Study setting** | **Sample Size** | **Study population** | **Case definition** | **Prevalence period** | **Prevalence** | **Risk of Bias Summary Score** |
| --- | --- | --- | --- | --- | --- | --- | --- | --- | --- | --- |
| Adamson (39) | 2006 | Scotland | Face-to-face questionnaire | Community | 858 | Three cohorts aged around 15, 35 and 55 years. Urban. Residents of Glasgow | Do you regularly suffer from any swelling, pain or stiffness in either shoulder. Marked on body map | Unclear | 25.52% | Moderate |
| Chard (28) | 1991 | England | Face-to-face questionnaire and physical examination | Community | 644 | Age 70+. Residents of Cambridge | Shoulder condition by physical examination | Point | 14.3% | Moderate |
| Choi (40) | 2013 | South Korea | Face-to-face questionnaire | Community | 1576 | Age 18+. No symptoms arising from rheumatic disorders and trauma | Pain caused by ADL including work activities, that continued for over a week in the previous year or at least once every month at a severe or extremely severe level according to the NIOSH criteria | 12 months | 16.9% | Low |
| Docking (41) | 2015 | Scotland | Face-to-face questionnaire | Community | 6013 | Age 55+. Registered in general practices | Any aches or pain that lasted for one day or longer | 1 month | 27% | Moderate |
| Duncan (42) | 2011 | England | Face-to-face questionnaire | Community | 1029 | Age 85+. Residents of Newcastle | Pain of the shoulder on most days during the last month | 1 month | 30.7% | Moderate |
| Hill (43) | 2010 | Australia | Face-to-face questionnaire | Community | 3488 | Age 18+. Rural. Residents of West Adelaide | pain or aching in the shoulder at rest or when moving, on most days for at least a month or stiffness in the shoulder when getting out of bed in the morning on most days for at least a month | Lifetime | 22.25% | Low |
| Lock (44) | 1999 | England | Postal questionnaire | Community | 1546 | Age 17+. Urban. Residents of Newcastle-upon-tyne | Shoulder problem which had restricted normal activities for more than 1 week | 12 months | 19.98% | Moderate |
| Salaffi (45) | 2005 | Italy | Face-to-Face questionnaire and physical examination | Community | 2155 | Age 18+. Residents of Marche region. Excluding dementia or mental illness | Shoulder tendinitis or adhesive capsulitis | Unclear | 3.06% | Moderate |
| Sansone (46) | 2014 | Italy | Face-to-face questionnaire | Community | 302 | Female cashiers only. Resident in Northern Italy | Pain in the shoulders for at least one day a month, or at least 7 consecutive days | 12 months | 19.90% | Low |
| Walker-Bone (47) | 2004 | England | Face-to-Face questionnaire and physical examination | Community | 9696 | Age 25-64. Southampton. Not suffering from illness or recent bereavement that made it inadvisable for them to be approached or impossible for them to answer a self-administered questionnaire | Symptoms of pain, numbness, or tingling in the shoulder lasting at least one day | 7 days | 7.4% | Low |
| Wright (48) | 2015 | USA | Face-to-face questionnaire | Community | 1697 | Age 45+. Non-institutionalized white and African American men and women resident in North-Carolina. | On most days do you have pain, aching, or stiffness of your left or right shoulders? | Unclear | 25.1% | Low |
| Urwin (49) | 1998 | England | Face-to-face questionnaire | Community | 5752 | Adults. Urban. Residents of Tameside and Glossop Area. Excluded if unsuitable for study as selected by GP (for example because of terminal illness | Pain in the area of the shoulder for more than one week | 1 month | 16% | Low |

**Studies defining shoulder pain using health records**

| **Study** | **Year** | **Country** | **Data ascertainment** | **Study setting** | **Sample Size** | **Study population** | **Case definition** | **Prevalence period** | **Prevalence** | **Risk of Bias Summary Score** |
| --- | --- | --- | --- | --- | --- | --- | --- | --- | --- | --- |
| Campbell (50) | 2016 | England | Electronic Medical Record | Primary care | 27014 | Age 30-74. Resident in North Staffordshire | Read codes for shoulder consultations | 12 months | 2.10% | Moderate |
| Greving (51) | 2012 | The Netherlands | Electronic Medical Record | Primary care | appr. 30000 | Age 18+. Registered with a practice and no history of shoulder pain | ICPC-coded L08 Shoulder Symptom/complaint + L92 Shoulder syndrome. | 12 months | 4.84% | Low |
| Jordan (52) | 2010 | UK | Electronic Medical Record | Primary care | 100758 | Residents of North Staffordshire | Read codes from GP records - musculoskeletal codes allocated to shoulder region | 12 months | 1.99% | Low |
| Kinge (53) | 2015 | Norway | Electronic Medical Record | Primary care | 4985870 | Age 15+. Non-institutionalized residents. | ICD-10 code M75 or ICPC-coded L08 Shoulder Symptom/complaint + L92 Shoulder syndrome. | 12 months | 4.37% | Moderate |
| Linsell (54) | 2006 | UK | Electronic Medical Record | Primary care | 658469 | Age 18+. Registered with a practice. | Read codes from GP records - musculoskeletal codes allocated to shoulder region | 12 months | 2.36% | Low |
| Swinkels (55) | 2014 | the Netherlands | Electronic Medical Record | Primary care | 1009083 |  | Shoulder complaints | 12 months | 3.15% | Low |
| Tekavec (56) | 2012 | Sweden | Electronic Medical Record | Primary care | 1169464 | Age 20+. Residents of Skane County | ICD-10 code M75 | 12 months | 1.01% | Moderate |

References

1. Al-Awadhi A.M., Olusi S.O., Moussa M., Shehab D., Al-Zaid N., Al-Herz A., et al. Musculoskeletal pain, disability and health-seeking behavior in adult Kuwaitis using a validated Arabic version of WHO-ILAR COPCORD Core Questionnaire. Clin Exp Rheumatol. 2004;22(2):177–83.

2. Cardiel M.H., Rojas-Serrano J. Community based study to estimate prevalence, burden of illness and help seeking behavior in rheumatic diseases in Mexico City. A COPCORD study. Clin Exp Rheumatol. 2002;20(5):617–24.

3. Chaaya M, Slim ZN, Habib RR, Arayssi T, Dana R, Hamdan O, et al. High burden of rheumatic diseases in Lebanon: a COPCORD study. Int J Rheum Dis. 2012;15(2):136–43.

4. Chopra A., Saluja M., Patil J., Tandale H.S. Pain and disability, perceptions and beliefs of a rural Indian population: A WHO-ILAR COPCORD study. J Rheumatol. 2002;29(3):614–21.

5. Dai S-M, Han X-H, Zhao D-B, Shi Y-Q, Liu Y, Meng J-M. Prevalence of rheumatic symptoms, rheumatoid arthritis, ankylosing spondylitis, and gout in Shanghai, China: a COPCORD study. J Rheumatol [Internet]. 2003 Oct;30(10):2245–51. Available from: https://www.ncbi.nlm.nih.gov/pubmed/14528524

6. F, Davatchi, A, Jamshidi, At B, J, Gholami, Mh F, M, Akhlaghi, et al. WHO-ILAR COPCORD study (stage 1, urban study) in Iran. J Rheumatol [Internet]. 2008;35(7):1384–90. Available from: http://search.ebscohost.com/login.aspx?direct=true&db=rzh&AN=105547604&site=ehost-live&scope=site&authtype=ip,shib&custid=s5040751

7. Davatchi F., Tehrani Banihashemi A., Gholami J., Faezi S.T., Forouzanfar M.H., Salesi M., et al. The prevalence of musculoskeletal complaints in a rural area in Iran: A WHO-ILAR COPCORD study (stage 1, rural study) in Iran. Clin Rheumatol. 2009;28(11):1267–74.

8. Del Río Nájera D, González-Chávez SA, Quiñonez-Flores CM, Peláez-Ballestas I, Hernández-Nájera N, Pacheco-Tena CF. Rheumatic Diseases in Chihuahua, México: A COPCORD Survey. JCR: Journal of Clinical Rheumatology [Internet]. 2016 Jun [cited 2021 Nov 5];22(4):188. Available from: https://journals.lww.com/jclinrheum/FullText/2016/06000/Rheumatic_Diseases_in_Chihuahua,_M_xico__A_COPCORD.5.aspx

9. Guevara-Pacheco S, Feicán-Alvarado A, Sanín LH, Vintimilla-Ugalde J, Vintimilla-Moscoso F, Delgado-Pauta J, et al. Prevalence of musculoskeletal disorders and rheumatic diseases in Cuenca, Ecuador: a WHO-ILAR COPCORD study. Rheumatol Int [Internet]. 2016 Sep;36(9):1195–204. Available from: http://dx.doi.org/10.1007/s00296-016-3446-y

10. Granados Y., Cedeno L., Rosillo C., Berbin S., Azocar M., Molina M.E., et al. Prevalence of musculoskeletal disorders and rheumatic diseases in an urban community in Monagas State, Venezuela: a COPCORD study. Clin Rheumatol [Internet]. 2015;34(5):871–7. Available from: http://link.springer.de/link/service/journals/10067/index.htm

11. Haq S.A., Darmawan J., Islam M.N., Uddin M.Z., Das B.B., Rahman F., et al. Prevalence of rheumatic diseases and associated outcomes in rural and urban communities in Bangladesh: A COPCORD study. J Rheumatol. 2005;32(2):348–53.

12. Joshi VL, Chopra A. Is there an urban-rural divide? Population surveys of rheumatic musculoskeletal disorders in the Pune region of India using the COPCORD Bhigwan model. J Rheumatol [Internet]. 2009 Mar;36(3):614–22. Available from: http://dx.doi.org/10.3899/jrheum.080675

13. Kolahi S., Khabbazi A., Malek Mahdavi A., Ghasembaglou A., Aminisani N., Somi M.H., et al. Prevalence of musculoskeletal disorders in Azar cohort population in Northwest of Iran. Rheumatol Int [Internet]. 2017;37(4):495–502. Available from: http://link.springer.de/link/service/journals/00296/index.htm

14. Londono J., Pelaez Ballestas I., Cuervo F., Angarita I., Giraldo R., Rueda J.C., et al. Prevalence of rheumatic disease in Colombia according to the Colombian Rheumatology Association (COPCORD) strategy. Prevalence study of rheumatic disease in Colombian population older than 18 years. Revista Colombiana de Reumatologia [Internet]. 2018;25(4):245–56. Available from: http://www.elsevier.com/locate/issn/01218123

15. Minh Hoa TT, Darmawan J, Chen SL, Van Hung N, Thi Nhi C, Ngoc An T. Prevalence of the rheumatic diseases in urban Vietnam: a WHO-ILAR COPCORD study. J Rheumatol [Internet]. 2003 Oct;30(10):2252–6. Available from: https://www.ncbi.nlm.nih.gov/pubmed/14528525

16. Nzambi JPD, Malemba JJ, Lebughe LP, Mpembele E, Mulenga C, Lukusa A, et al. The epidemiology of rheumatic disorders in a rural area of the Democratic Republic of Congo (DRC): A COPCORD study. African Journal of Rheumatology [Internet]. 2017 [cited 2021 Nov 5];5(2):64–9. Available from: https://www.ajol.info/index.php/ajr/article/view/158565

17. Quintana R, Silvestre AMR, Goñi M, García V, Mathern N, Jorfen M, et al. Prevalence of musculoskeletal disorders and rheumatic diseases in the indigenous Qom population of Rosario, Argentina. Clin Rheumatol [Internet]. 2016 Jul;35 Suppl 1:5–14. Available from: http://dx.doi.org/10.1007/s10067-016-3192-2

18. Reyes Llerena GA, Guibert Toledano M, Hernandez Martinez AA, Gonzalez Otero ZA, Alcocer Varela J, Cardiel MH, et al. Prevalence of musculoskeletal complaints and disability in Cuba. A community-based study using the COPCORD core questionnaire. Clin Exp Rheumatol. 2000;18(6):739–42.

19. Rodriguez-Amado J, Peláez-Ballestas I, Sanin LH, Esquivel-Valerio JA, Burgos-Vargas R, Pérez-Barbosa L, et al. Epidemiology of rheumatic diseases. A community-based study in urban and rural populations in the state of Nuevo Leon, Mexico. J Rheumatol Suppl [Internet]. 2011 Jan;86:9–14. Available from: http://dx.doi.org/10.3899/jrheum.100952

20. Sandoughi M., Zakeri Z., Tehrani Banihashemi A., Davatchi F., Narouie B., Shikhzadeh A., et al. Prevalence of musculoskeletal disorders in southeastern Iran: A WHO-ILAR COPCORD study (stage 1, urban study). Int J Rheum Dis. 2013;16(5):509–17.

21. Sarakbi H.A., Alsaed O., Hammoudeh M., Lutf A., Poil A.R., Ziyada A., et al. Epidemiology of musculoskeletal complaints and diseases in Qatar: A cross-sectional study. Qatar Medical Journal [Internet]. 2020;2020(2):29. Available from: https://www.qscience.com/content/journals/10.5339/qmj.2020.29

22. Veerapen K, Wigley RD, Valkenburg H. Musculoskeletal pain in Malaysia: a COPCORD survey. J Rheumatol [Internet]. 2007 Jan;34(1):207–13. Available from: https://www.ncbi.nlm.nih.gov/pubmed/17216688

23. Vega-Hinojosa O, Cardiel MH, Ochoa-Miranda P. Prevalence of musculoskeletal manifestations and related disabilities in a Peruvian urban population living at high altitude. COPCORD Study. Stage I. Reumatol Clin [Internet]. 2018 Sep;14(5):278–84. Available from: http://dx.doi.org/10.1016/j.reuma.2017.01.011

24. Antonopoulou M.D., Alegakis A.K., Hadjipavlou A.G., Lionis C.D. Studying the association between musculoskeletal disorders, quality of life and mental health. A primary care pilot study in rural Crete, Greece. BMC Musculoskelet Disord. 2009;10(1):143.

25. Badcock L.J., Lewis M., Hay E.M., McCarney R., Croft P.R. Chronic shoulder pain in the community: A syndrome of disability or distress? Ann Rheum Dis. 2002;61(2):128–31.

26. Bento TPF, Genebra CV dos S, Cornélio GP, Biancon RDB, Simeão S, Penteado ra FA, et al. Prevalence and factors associated with shoulder pain in the general population: a cross-sectional study. Fisioterapia e Pesquisa [Internet]. 2019;26(4):401–6. Available from: http://search.ebscohost.com/login.aspx?direct=true&db=rzh&AN=141053485&site=ehost-live&scope=site&authtype=ip,shib&custid=s5040751

27. Bingefors K., Isacson D. Epidemiology, co-morbidity, and impact on health-related quality of life of self-reported headache and musculoskeletal pain - A gender perspective. Eur J Pain. 2004;8(5):435–50.

28. Chard M.D., Hazleman R., Hazleman B.L., King R.H., Reiss B.B. Shoulder disorders in the elderly: A community survey. Arthritis Rheum. 1991;34(6):766–9.

29. Engebretsen KB, Grotle M, Natvig B. Patterns of shoulder pain during a 14-year follow-up: results from a longitudinal population study in Norway. Shoulder & elbow. 2015;7(1):49–59.

30. C, Ihlebæk, Hr E, H, Ursin. Prevalence of subjective health complaints (SHC) in Norway. Scand J Public Health [Internet]. 2002;30(1):20–9. Available from: http://search.ebscohost.com/login.aspx?direct=true&db=rzh&AN=106813277&site=ehost-live&scope=site&authtype=ip,shib&custid=s5040751

31. Jacobs RJ, Collias BA, Rana AM, Wallace EM, Kane MN, Boesler DR. Identification of musculoskeletal disorders in medically underserved regions of South America and Vietnam. JAOA: Journal of the American Osteopathic Association [Internet]. 2015;115(1):12–22. Available from: http://search.ebscohost.com/login.aspx?direct=true&db=rzh&AN=109691143&site=ehost-live&scope=site&authtype=ip,shib&custid=s5040751

32. Minaur N., Sawyers S., Parker J., Darmawan J. Rheumatic Disease in an Australian Aboriginal Community in North Queensland, Australia. A WHO-ILAR COPCORD Survey. J Rheumatol. 2004;31(5):965–72.

33. Patel K.V., Guralnik J.M., Dansie E.J., Turk D.C. Prevalence and impact of pain among older adults in the United States: Findings from the 2011 National Health and Aging Trends Study. Pain [Internet]. 2013;154(12):2649–57. Available from: http://www.elsevier.com/locate/painonline

34. Picavet HSJ, Schouten JSAG. Musculoskeletal pain in the Netherlands: prevalences, consequences and risk groups, the DMC(3)-study. Pain [Internet]. 2003;102(1):167–78. Available from: http://search.ebscohost.com/login.aspx?direct=true&db=rzh&AN=104731047&site=ehost-live&scope=site&authtype=ip,shib&custid=s5040751

35. Rechardt M, Shiri R, Karppinen J, Jula A, Heliövaara M, Viikari-Juntura E. Lifestyle and metabolic factors in relation to shoulder pain and rotator cuff tendinitis: a population-based study. BMC Musculoskelet Disord [Internet]. 2010 Jul 20;11:165. Available from: http://dx.doi.org/10.1186/1471-2474-11-165

36. Sanchez Santos MT, Williamson E, Bruce J, Ward L, Mallen CD, Garrett A, et al. Cohort profile: Oxford Pain, Activity and Lifestyle (OPAL) Study, a prospective cohort study of older adults in England. BMJ Open [Internet]. 2020 Sep 3;10(9):e037516. Available from: http://dx.doi.org/10.1136/bmjopen-2020-037516

37. Thielke SM, Whitson H, Diehr P, O’Hare A, Kearney PM, Chaudhry SI, et al. Persistence and Remission of Musculoskeletal Pain in Community-Dwelling Older Adults: Results from the Cardiovascular Health Study. J Am Geriatr Soc [Internet]. 2012;60(8):1393–400. Available from: http://search.ebscohost.com/login.aspx?direct=true&db=rzh&AN=104361314&site=ehost-live&scope=site&authtype=ip,shib&custid=s5040751

38. Vindigni D, Griffen D, Perkins J, Da Costa C, Parkinson L. Prevalence of musculoskeletal conditions, associated pain and disability and the barriers to managing these conditions in a rural, Australian Aboriginal community. Rural Remote Health. 2004;4(3):230.

39. Adamson J, Ebrahim S, Dieppe P, Hunt K. Prevalence and risk factors for joint pain among men and women in the West of Scotland Twenty-07 study. Ann Rheum Dis. 2006;520–4.

40. Choi K., Park J.-H., Cheong H.-K. Prevalence of musculoskeletal symptoms related with activities of daily living and contributing factors in Korean adults. J Prev Med Public Health [Internet]. 2013;46(1):39–49. Available from: http://synapse.koreamed.org/Synapse/Data/PDFData/0056JPMPH/jpmph-46-39.pdf

41. Docking R.E., Beasley M., Steinerowski A., Jones E.A., Farmer J., Macfarlane G.J., et al. The epidemiology of regional and widespread musculoskeletal pain in rural versus urban settings in those >=55 years. British Journal of Pain [Internet]. 2015;9(2):86–95. Available from: http://bjp.sagepub.com/

42. Duncan R., Francis R.M., Collerton J., Davies K., Jagger C., Kingston A., et al. Prevalence of arthritis and joint pain in the oldest old-findings from the Newcastle 85+ study. Age Ageing. 2011;40.

43. Hill C.L., Gill T.K., Shanahan E.M., Taylor A.W. Prevalence and correlates of shoulder pain and stiffness in a population-based study: The North West Adelaide Health Study. Int J Rheum Dis. 2010;13(3):215–22.

44. C, Lock, V, Allgar, K, Jones, G, Marples, C, Ch, ler, et al. Prevalence of back, neck and shoulder problems in the inner city: implications for the provision of physiotherapy services in primary healthcare. Physiother Res Int [Internet]. 1999;4(3):161–9. Available from: http://search.ebscohost.com/login.aspx?direct=true&db=rzh&AN=107231297&site=ehost-live&scope=site&authtype=ip,shib&custid=s5040751

45. Salaffi F, De Angelis R, Grassi W, Prevalence MP, Study IG (mapping). Prevalence of musculoskeletal conditions in an Italian population sample: results of a regional community-based study. I. The MAPPING study. Clin Exp Rheumatol. 2005;23(6):819–28.

46. Sansone V, Bonora C, Boria P, Meroni R. Women performing repetitive work: is there a difference in the prevalence of shoulder pain and pathology in supermarket cashiers compared to the general female population? Int J Occup Med Environ Health [Internet]. 2014;27(5):722–35. Available from: http://search.ebscohost.com/login.aspx?direct=true&db=rzh&AN=109681503&site=ehost-live&scope=site&authtype=ip,shib&custid=s5040751

47. Walker-Bone K., Palmer K.T., Reading I., Coggon D., Cooper C. Prevalence and impact of musculoskeletal disorders of the upper limb in the general population. Arthritis Care Res. 2004;51(4):642–51.

48. Wright A.R., Shi X.A., Busby-Whitehead J., Jordan J.M., Nelson A.E. The prevalence of neck and shoulder symptoms and associations with comorbidities and disability: The Johnston county osteoarthritis project. MYOPAIN [Internet]. 2015;23(1):34–44. Available from: http://www.tandfonline.com/loi/imup21

49. Urwin M, Symmons D, Allison T, Brammah T, Busby H, Roxby M, et al. Estimating the burden of musculoskeletal disorders in the community: the comparative prevalence of symptoms at different anatomical sites, and the relation to social deprivation. Ann Rheum Dis. 1998;57(11):649–55.

50. Campbell P, Shraim M, Jordan KP, Dunn KM. In sickness and in health: A cross-sectional analysis of concordance for musculoskeletal pain in 13,507 couples. Eur J Pain [Internet]. 2016;20(3):438–46. Available from: http://search.ebscohost.com/login.aspx?direct=true&db=rzh&AN=112968676&site=ehost-live&scope=site&authtype=ip,shib&custid=s5040751

51. Greving K., Dorrestijn O., Winters J.C., Groenhof F., Van Der Meer K., Stevens M., et al. Incidence, prevalence, and consultation rates of shoulder complaints in general practice. Scand J Rheumatol. 2012;41(2):150–5.

52. Jordan KP, Kadam UT, Hayward R, Porcheret M, Young C, Croft P. Annual consultation prevalence of regional musculoskeletal problems in primary care: an observational study. BMC Musculoskelet Disord [Internet]. 2010 Jul 2;11(1):144. Available from: http://dx.doi.org/10.1186/1471-2474-11-144

53. Kinge J.M., Knudsen A.K., Skirbekk V., Vollset S.E. Musculoskeletal disorders in Norway: Prevalence of chronicity and use of primary and specialist health care services. BMC Musculoskelet Disord [Internet]. 2015;16(1):75. Available from: http://www.biomedcentral.com/bmcmusculoskeletdisord/

54. Linsell L., Dawson J., Zondervan K., Rose P., Randall T., Fitzpatrick R., et al. Prevalence and incidence of adults consulting for shoulder conditions in UK primary care; patterns of diagnosis and referral. Rheumatology. 2006;45(2):215–21.

55. S. Swinkels IC, Kooijman MK, Spreeuwenberg PM, Bossen D, Leemrijse CJ, van Dijk CE, et al. An Overview of 5 Years of Patient Self-Referral for Physical Therapy in the Netherlands. Phys Ther [Internet]. 2014;94(12):1785–95. Available from: http://search.ebscohost.com/login.aspx?direct=true&db=rzh&AN=103922816&site=ehost-live&scope=site&authtype=ip,shib&custid=s5040751

56. Tekavec E, Joud A, Rittner R, Mikoczy Z, Nord, Er C, et al. Population-based consultation patterns in patients with shoulder pain diagnoses. BMC Musculoskelet Disord. 2012;
