# Supplementary material for: A systematic review of the global prevalence and incidence of shoulder pain
Source: BMC Musculoskelet Disord. 2022 Dec 8;23:1073. doi: 10.1186/s12891-022-05973-8 (PMC9730650; doi:10.1186/s12891-022-05973-8)
Supplement: Supplementary file 2 — Additional file 2: Supplementary Table 2. Study characteristics and results of incidence studies [file 12891_2022_5973_MOESM2_ESM.docx]

Table 2. Summary of incidence studies

| **Study** | **Year** | **Country** | **Data ascertainment** | **Study setting** | **Sample size** | **Study population** | **Case definition** | **Incidence period** | **Incidence** | **Risk of Bias Summary Score** |
| --- | --- | --- | --- | --- | --- | --- | --- | --- | --- | --- |
| Al-Awadhi [[2]](https://paperpile.com/c/vhvqSR/TvFm) | 2005 | Kuwait | Face-to-face questionnaire | Community | 3341 | Age 18+, Kuwaiti nationals | Pain, stiffness or swelling in the shoulder region. Marked on body map | >one year | 11.4 per 1000 person-years | Low |
| Bot [[3]](https://paperpile.com/c/vhvqSR/yXTY) | 2005 | Netherlands | Electronic Medical Record | Primary care | 375899 | Age 0-80+ | ICPC-coded L08 Shoulder Symptom/complaint + L92 Shoulder syndrome. | if GP considered it as being separate from earlier problems | 23.1 per 1000 person-years | Low |
| Feleus [[1]](https://paperpile.com/c/vhvqSR/edK4) | 2008 | Netherlands | Electronic Medical Record | Primary care | 32775 | Age 18-64 | ICPC-coded L08 Shoulder Symptom/complaint + L92 Shoulder syndrome. | >6 months | 29.5 per 1000 person-years | Low |
| Greving [[4]](https://paperpile.com/c/vhvqSR/Dyu5) | 2012 | Netherlands | Electronic Medical Record | Primary care | appr. 30000 | Age 18+. Registered with a practice and no history of shoulder pain | ICPC-coded L08 Shoulder Symptom/complaint + L92 Shoulder syndrome. | No history of shoulder pain | 29.3 per 1000 person-years | Low |
| Haq [[5]](https://paperpile.com/c/vhvqSR/eMDF) | 2008 | Bangladesh | Face-to-face questionnaire | Community | 2685 | Age 15+. Residents of six villages near Dhaka | Pain, stiffness or swelling in the shoulder region. Marked on body map | >14 days | 62 per 1000 person-years | Low |
| Linsell [[6]](https://paperpile.com/c/vhvqSR/w505) | 2006 | UK | Electronic Medical Record | Primary care | 658469 | Age 18+. Registered with a practice. | Read codes from GP records - musculoskeletal codes allocated to shoulder region | >3 years | 14.7 per 1000 person-years | Low |
| Tekavec [[7]](https://paperpile.com/c/vhvqSR/3M1H) | 2012 | Sweden | Electronic Medical Record | Primary care | 1169464 | Age 20+. Residents of Skane County | ICD-10 code M75 | >2 year | 7.7 per 1000 person-years | Moderate |
| Windt [[8]](https://paperpile.com/c/vhvqSR/wbX3) | 1995 | Netherlands | Electronic Medical Record | Primary care | 35150 | Age 18+ | Intrinsic shoulder complaints, origination from within the shoulder joint | >1 year | 14.7 per 1000 person-years | Low |

References

[1] [A, Feleus, Sma B-Z, Hs M, Rmd B, Jan V, Bw K. Incidence of non-traumatic complaints of arm, neck and shoulder in general practice. Man Ther 2008;13:426–433. Available:](http://paperpile.com/b/vhvqSR/edK4) <http://search.ebscohost.com/login.aspx?direct=true&db=rzh&AN=105693530&site=ehost-live&scope=site&authtype=ip,shib&custid=s5040751.>

[2] [Al-Awadhi AM, Olusi SO, Al-Saeid K, Moussa M, Shehab D, Al-Zaid N, Al-Herz A, Al-Jarallah K. Incidence of musculoskeletal pain in adult Kuwaitis using the validated Arabic version of the WHO-ILAR COPCORD Core Questionnaire. Ann Saudi Med 2005;25:459–462. doi:](http://paperpile.com/b/vhvqSR/TvFm)[10.5144/0256-4947.2005.459](http://dx.doi.org/10.5144/0256-4947.2005.459)[.](http://paperpile.com/b/vhvqSR/TvFm)

[3] [Bot SDM, van der Waal JM, Terwee CB, van der Windt D, Schellevis FG, Bouter LM, Dekker J. {Incidence and prevalence of complaints of the neck and upper extremity in general practice}. Ann Rheum Dis 2005:{118–123}.](http://paperpile.com/b/vhvqSR/yXTY)

[4] [Greving K., Dorrestijn O., Winters J.C., Groenhof F., Van Der Meer K., Stevens M., Diercks R.L. Incidence, prevalence, and consultation rates of shoulder complaints in general practice. Scand J Rheumatol 2012;41:150–155.](http://paperpile.com/b/vhvqSR/Dyu5)

[5] [Haq S.A., Darmawan J., Islam N., Ahmed M., Banik S.K., Fazlur Rahman A.K.M., Alam M.N., Tahir M., Rasker J.J. Incidence of musculoskeletal pain and rheumatic disorders in a Bangladeshi rural community: A WHO-APLAR-COPCORD study. Int J Rheum Dis 2008;11:216–223.](http://paperpile.com/b/vhvqSR/eMDF)

[6] [Linsell L., Dawson J., Zondervan K., Rose P., Randall T., Fitzpatrick R., Carr A. Prevalence and incidence of adults consulting for shoulder conditions in UK primary care; patterns of diagnosis and referral. Rheumatology 2006;45:215–221.](http://paperpile.com/b/vhvqSR/w505)

[7] [Tekavec E, Joud A, Rittner R, Mikoczy Z, Nord, Er C, Petersson IF, Englund M. {Population-based consultation patterns in patients with shoulder pain diagnoses}. BMC Musculoskelet Disord 2012.](http://paperpile.com/b/vhvqSR/3M1H)

[8] [Van der Windt D.A.W.M., Koes B.W., De Jong B.A., Bouter L.M. Shoulder disorders in general practice: Incidence, patient characteristics, and management. Ann Rheum Dis 1995;54:959–964.](http://paperpile.com/b/vhvqSR/wbX3)
