# Supplementary material for: A systematic review of the global prevalence and incidence of shoulder pain
Source: BMC Musculoskelet Disord. 2022 Dec 8;23:1073. doi: 10.1186/s12891-022-05973-8 (PMC9730650; doi:10.1186/s12891-022-05973-8)
Supplement: Supplementary file 3 — Additional file 3: Supplementary Fig. 1. Database search strategy [file 12891_2022_5973_MOESM3_ESM.pdf]

1. exp Shoulder Joint/
2. shoulder*.ti,ab,kf.
3. acromioclavic*.ti,ab,kf.
4. glenohumer*.ti,ab,kf.
5. subacromia*.ti,ab,kf.
6. or/1-5
7. exp Pain/
8. pain*.ti,ab,kf.
9. 7 or 8
10. 6 and 9
11. exp Shoulder Pain/
12. Shoulder Impingement Syndrome/
13. exp Bursitis/
14. Rotator Cuff/
15. (shoulder adj3 (problem* or complaint* or syndrome* or symptom* or disorder* or bursitis)).ti,ab,kf.
16. adhesive capsuliti*.ti,ab,kf.
17. frozen shoulder*.ti,ab,kf.
18. or/10-17
19. Incidence/
20. Prevalence/
21. Epidemiology/
22. incidence.ti,ab,kf.
23. prevalence.ti,ab,kf.
24. epidemiolog*.ti,ab,kf.
25. occurrence.ti,ab,kf.
26. "disease frequenc*".ti,ab,kf.
27. exp Cross-Sectional Studies/
28. exp Cohort Studies/
29. cross-section*.ti,ab,kf.
30. cohort*.ti,ab,kf.
31. survey*.ti,ab,kf.
32. or/19-31
33. exp Family Practice/
34. exp Physicians, Family/
35. exp Primary Health Care/
36. exp Community Health Services/
37. "general practi*".ti,ab,kf.
38. (primary adj2 care*).ti,ab,kf.
39. "family practi*".ti,ab,kf.
40. "family physician*".ti,ab,kf.
41. "family doctor*".ti,ab,kf.
42. GP.ti,ab,kf.
43. communit*.ti,ab,kf.
44. general population.ti,ab,kf.
45. countr*.ti,ab,kf.
46. (national or nationwide).ti,ab,kf.

47. or/33-46
48. 18 and 32 and 47
