# Supplementary figures and images for: A systematic review of the global prevalence and incidence of shoulder pain
Source: BMC Musculoskelet Disord. 2022 Dec 8;23:1073. doi: 10.1186/s12891-022-05973-8 (PMC9730650; doi:10.1186/s12891-022-05973-8)

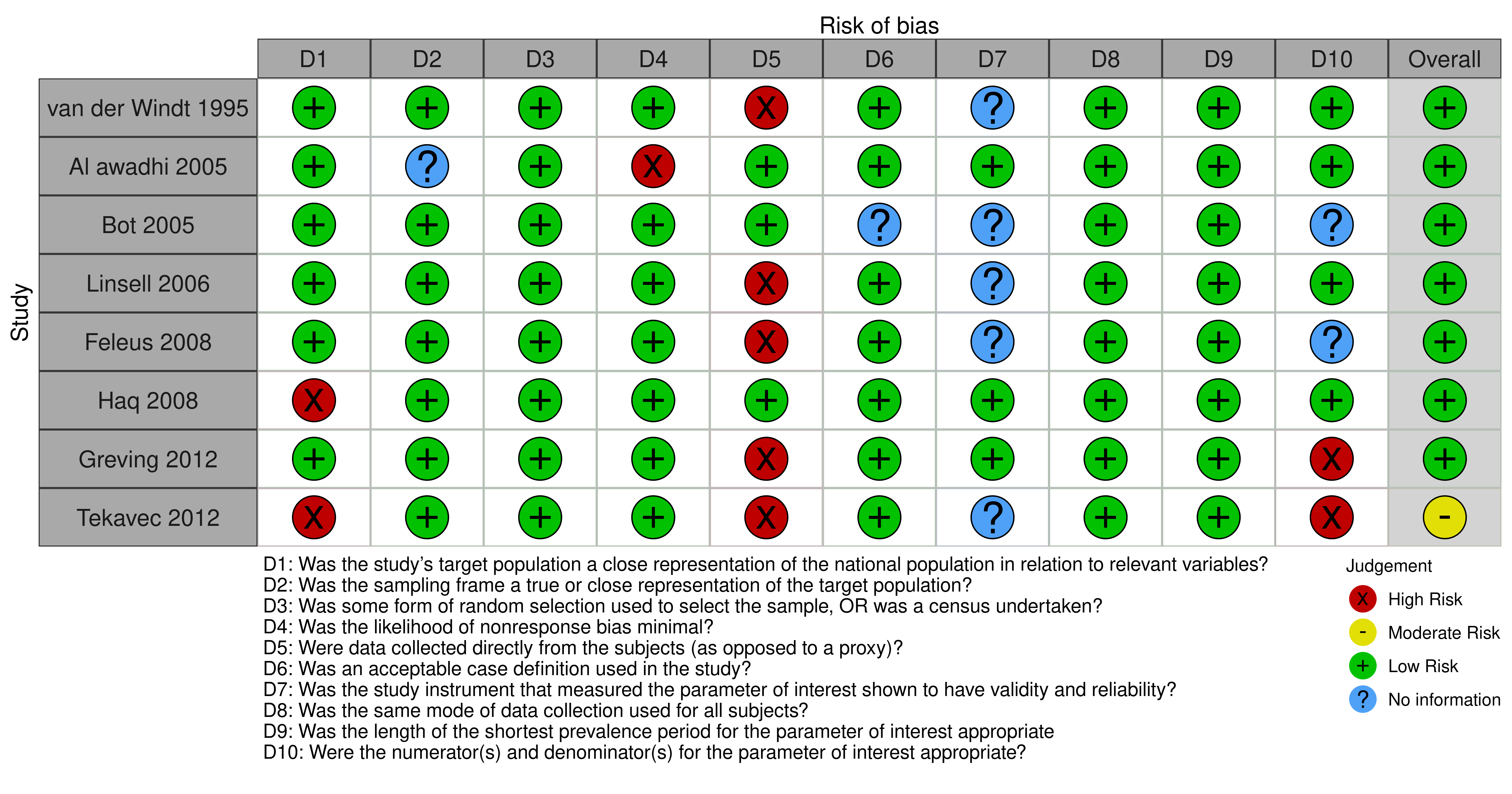

Supplement: Supplementary file 5 — Additional file 5: Supplementary Fig. 3. Risk of bias scores incidence studies [file 12891_2022_5973_MOESM5_ESM.png]
